# Evidence‐based practice integration in hospital wards—The complexities and challenges in achieving evidence‐based practice in clinical nursing

**DOI:** 10.1002/nop2.259

**Published:** 2019-03-18

**Authors:** Åste Renolen, Esther Hjälmhult, Sevald Høye, Lars Johan Danbolt, Marit Kirkevold

**Affiliations:** ^1^ Institute of Health and Society University of Oslo Oslo Norway; ^2^ Department of Medicine Innlandet Hospital Trust Lillehammer Norway; ^3^ Centre for Evidence‐Based Practice Western Norway University of Applied Sciences Bergen Norway; ^4^ Faculty of Public Health Inland Norway University of Applied Sciences Elverum Norway; ^5^ Centre of Psychology of Religion Innlandet Hospital Trust Ottestad Norway; ^6^ Norwegian School of Theology Oslo Norway

**Keywords:** clinical practice guidelines, evidence‐based practice, hospital, huddle board, implementation, nurses, research utilization, whiteboard

## Abstract

**Aim:**

Exploring the processes involved in two different strategies to integrate evidence‐based practice into nursing practice.

**Design:**

Classical grounded theory methodology was used.

**Methods:**

Data were collected through 90 hr of observation and 4 focus groups among clinical nurses in two different hospital wards.

**Results:**

We identified a multidimensional evidence‐based practice integration framework that illuminates the complexities involved in the integration process. The dimensions were approaches to evidence‐based practice, positions of evidence‐based practice and levels of evidence‐based practice. The interactions between the dimensions gave five combinations; an explicit evidence‐based practice performed as a parallel to daily work at the systems level, an implicit evidence‐based practice integrated into daily work at the systems level, an explicit evidence‐based practice integrated into daily work at the individual level, an explicit evidence‐based practice integrated into daily work at the systems level and an implicit evidence‐based practice integrated into daily work at the individual level.

## 1 INTRODUCTION

Huge amounts of relevant research evidence exist in health and nursing sciences, which is not integrated into clinical practice due to translation and implementation challenges (Greenhalgh, 2018; Grimshaw, Eccles, Lavis, Hill, & Squires, 2012; Song et al., 2010). A large number of the studies have aimed to identify factors that facilitate or hinder the integration of new research evidence into the nursing practice (Cochrane et al., 2007; Estabrooks, Floyd, Scott‐Findlay, O'Leary, & Gushta, 2003; Funk, Champagne, Wiese, & Tornquist, 1991; Sadeghi‐Bazargani, Tabrizi, & Azami‐Aghdash, 2014; Solomons & Spross, 2011). However, few studies have investigated the actual processes of attempting to integrate evidence‐based practice (EBP) into daily practice, which was the purpose of this study. In the research literature, there has been an inconsistent use of terminologies regarding implementation of new practices (Damschroder et al., 2009; May & Finch, 2009). In this paper, we use the concept of implementation to mean organizing the adoption of EBP in organizational units, while integration refers to the routinizing and sustaining of new practices.

### 1.1 Background

EBP implies the integration of clinical expertise with systematically obtained research evidence, considering resources available and patient preferences in each patient situation (DiCenso, Guyatt, & Ciliska, 2005; Polit & Beck, 2016; Sackett, Rosenberg, Gray, Haynes, & Richardson, 1996). It may be regarded as a strategy or a general way of thinking aimed at achieving the best treatment and care in each individual patient situation. Furthermore, EBP also involves organizational activities such as integrating research evidence through the development of evidence‐based (EB) guidelines (Polit & Beck, 2016).

The implementation of research evidence has been challenging in nursing practice, and we need more knowledge regarding how to translate research into daily health and nursing care (Kajermo et al., 2010; Mallion & Brooke, 2016; Squires et al., 2011). Clinical nurses seem to value personal experience together with information learned in nursing school and information from colleagues as their most important source of knowledge, rather than basing practice on current research evidence (Adib‐Hajbaghery, 2007; Bischoff & Hinojosa, 2013; Renolen & Hjälmhult, 2015; Yoder et al., 2014). An association between higher reported levels of emotional exhaustion and lower reported levels of research use has been affirmed (Estabrooks, Midodzi, Cummings, & Wallin, 2007). As well, a more favourable context related to culture, good leadership and recognition for a job well done has resulted in higher research use (Estabrooks et al., 2007). In each culture, particular ideas or activities may be more valued than others (Scott‐Findlay & Golden‐Biddle, 2005). In a ward culture characterized by engagement in EBP and quality improvement, leadership and clinicians may to a greater extent succeed in changing practice (Saunders & Vehviläinen‐Julkunen, 2017). A ward culture characterized by rigid completion of practical tasks rather than engagement in EBP may not easily facilitate opportunities for research use or for changing practice (Henderson, Cooke, Creedy, & Walker, 2012; Ryan, 2016). Furthermore, promoting research use in an environment characterized by work overload among nurses and lack of teamwork structure that facilitate research use, may be demanding (Solomons & Spross, 2011). Studies have indicated that healthcare workers describe a change in practice as hard work and that continuing with the existing practice in daily work with an already huge workload is less demanding (Asadoorian, Hearson, Satyanarayana, & Ursel, 2010; Fink, Thompson, & Bonnes, 2005).

The potential for achieving practice changes through adopting EBP depends on the interaction between the characteristics of the evidence, the clinicians and the context of practice in the healthcare setting (Greenhalgh, Robert, Macfarlane, Bate, & Kyriakidou, 2004). It occurs as a complex process where people—often through dialogue with others—are active participants in innovations and which research must address (Greenhalgh, 2018; Greenhalgh et al., 2004).

In this study, we investigated the integration of EBP in clinical practice in hospital wards by studying in depth two different methods applied by clinicians. One method involved nurses working with an EBP project to develop local clinical guidelines. The other method included integrating EBP/EB guidelines through an interdisciplinary use of huddle board sessions.

### 1.2 Aim

The aim of this study was to explore the processes involved in two different strategies applied to integrate EBP to understand the complexities and challenges in clinical nurses' daily work better when they attempt to integrate EBP.

## 2 THE STUDY

### 2.1 Design

The data used in this study were collected and analysed through classical grounded theory methodology (Glaser, 1978, 1998; Glaser & Strauss, 1967). In grounded theory, the researcher initially has an open, inductive approach to data by systematically collecting the data from practice. As codes and categories emerge, one introduces a more focused approach to explore relationships between different properties in codes and categories, based on hypotheses formulated from the data analysis in the initial phase (Glaser, 1978, 1998).

### 2.2 Methods

#### 2.2.1 Setting and participants

The study was conducted in a Norwegian hospital trust consisting of six somatic hospitals scattered over a wide geographical area. Data were collected in two medical wards treating patients with different diagnoses in two different geographical locations eight to nine years after the hospital trust introduced EBP with the purpose of enhancing competence among health professionals (Vandvik & Eiring, 2011). According to grounded theory, wards, research methods, participants and situations were selected through theoretical sampling (Glaser & Strauss, 1967). Ward A was chosen based on the ward's engagement in an EBP project, initially guided by a general perspective and problem area. Ward B was included as it was assumed to be able to contribute information to fortify the emerging codes and categories in the theory development (Glaser, 1978; Glaser & Strauss, 1967). The participating wards and nurses are presented in Table 1.

**Table 1:** The participating wards and nurses

	Ward A	Ward B	Total
Number of beds	18 patient beds	38 patient beds	
Working groups	2 working groups	4 working groups, of whom 2 groups were participating	
Staff	33 nurses	63 nurses	96 nurses
	3 assistants	5 assistants	8 assistants
Hours of observations	36 hr	54 hr	90 hr
Number of observed nurses^a^	28 nurses	35 nurses	63 nurses
Focus groups	2	2	4
Nurses participating in focus groups (from the population of observed nurses)	10 nurses	8 nurses	18 nurses

a The nurses (*N* = 63): 39 registered nurses with a bachelor's degree awarded after 3 years of university‐level education, 9 assistant nurses with two years of upper secondary education. Of the remaining 15 nurses, two had a master's degree and 13 had twelve‐ to eighteen‐month specializations after their bachelor's degree. The types of specialization were relevant for the wards (here without a further specification to ensure anonymity).

**Figure 1:**
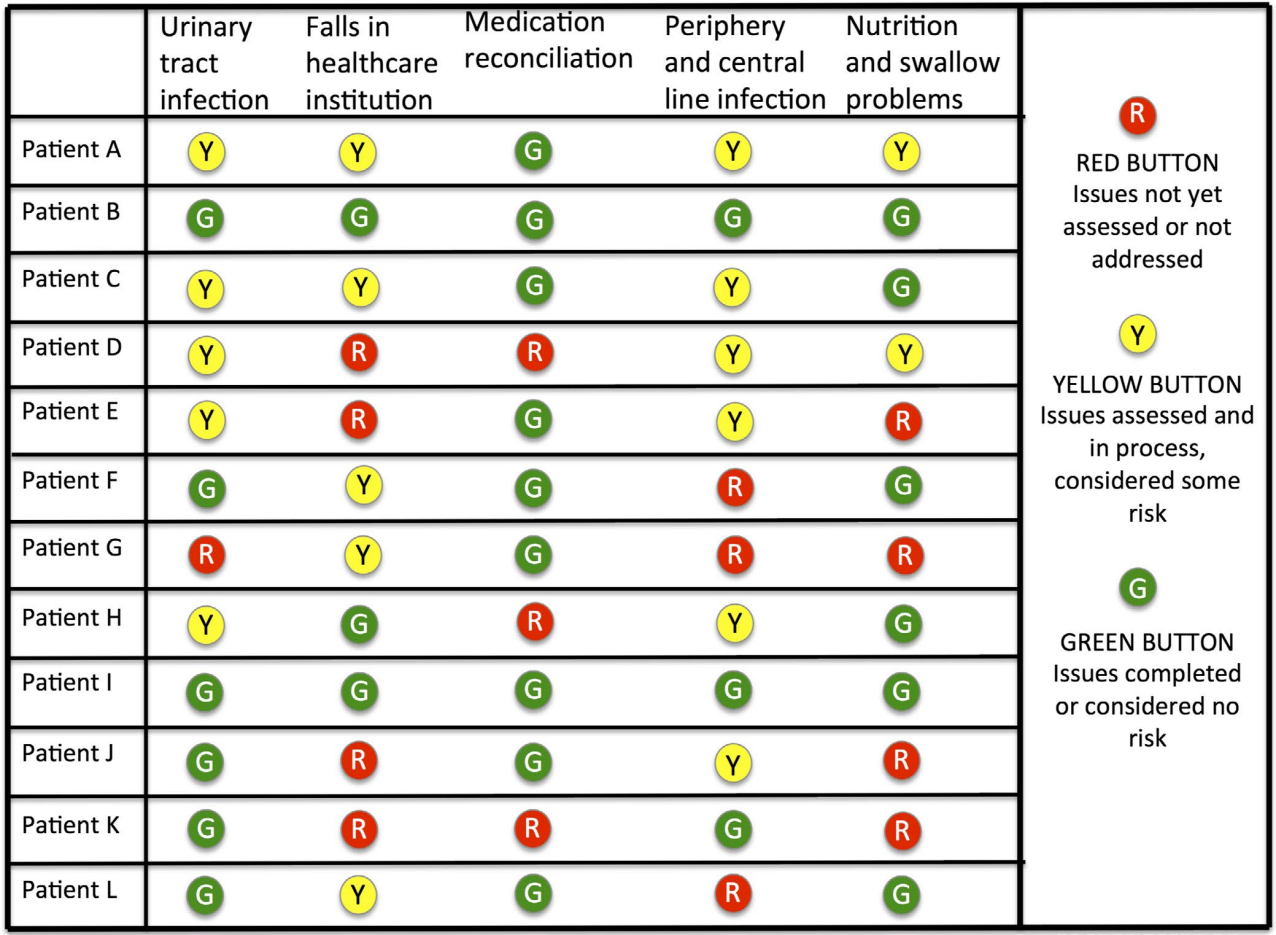
Example of a risk assessment huddle board

Ward B was using a huddle board to improve clinical practice and reduce patient harm in clinical practice. Huddles are short structural meetings among interdisciplinary healthcare workers (Glymph et al., 2015). Huddle board is a whiteboard used in a huddle as a visual patient risk assessment tool (Figure 1) introducing EB guidelines in daily work. Further information about Ward A and Ward B is outlined in Boxes.

BOX 1 **Ward A—Participating in an EBP project**

1 In Ward A, most nurses participated in an EBP project that had been ongoing for approximately two years. They were working in groups to find new evidence and to develop and implement clinical EB guidelines with the purpose of improving patient treatment and care. The project manager together with a teaching nurse allocated funds from the hospital to enable the nurses to participate in groups by obtaining dedicated time for this work. The nurses participated voluntarily in four different groups that worked one at a time, each with a self‐determined theme. To a various degree, the nurses were knowledgeable regarding asking and formulating questions, literature search, critical appraisal, applying new knowledge and evaluation. The groups worked to summarize the literature/work and planned to write up the process and results on internal teaching days and when they could find time for it.

BOX 2 **Ward B—Integrating a patient safety huddle board programme**

1 The employees in Ward B had a daily focus on quality improvement and had participated in different small EBP projects. When data collection started, the ward was in an early phase of integrating a huddle board programme initiated by the hospital leadership aiming to improve clinical practice and reduce patient harm. The initiative was anchored in the Norwegian Patient Safety Programme, where a group of healthcare experts identified several target areas with recommendations and measures based on the current available evidence, such as systematic reviews and national clinical practice guidelines (Norwegian Ministry of Health & Care Services, 2015). Locally, each ward was assigned target areas determined by the hospital leadership, with some also chosen by the physicians and nurses in the ward. A project manager in the hospital leadership decided which guidelines to locally tailor and implement in each working team through interdisciplinary daily meetings (i.e. “huddles”). The clinicians were supposed to use the EB guidelines together with their expertise, available resources and patient preferences in EBP performance. A template for checking off and scoring the patients informed by the actual guideline for each target area was used.

#### 2.2.2 Data collection

Data were collected between March 2014 and November 2015. The lead researcher was a nurse employed at one of the hospitals where the study was conducted. The researcher therefore knew the organization, general routines, quality improvement measures and the system of clinical guidelines. However, at the time of the study, she was acting in a researcher role. The researcher mapped out the EBP activities in the relevant hospital wards, excluding wards well known to her. The data collection began with participant observation in Ward A, providing the opportunity to study the nurses' behaviour in relation to their attempts to integrate EBP while continuing to conduct their daily work in the ward (Creswell, 2013; Polit & Beck, 2016). The researcher wrote descriptive and reflective field notes during the observations and directly afterwards (Creswell, 2013). On finishing the observations and its analyses in Ward A, two focus groups were held to give the observed nurses an opportunity to discuss their concerns and to bring up questions that had emerged from the collected data (Polit & Beck, 2016). A thematic interview guide was used, starting with an open question about the nurses' experiences with EBP. In line with grounded theory methodology, we stayed open and let the participants talk about their concerns (Glaser, 2013). Afterwards, data were collected in the same way in Ward B. Based on emerging codes and categories, ward B was chosen because they attempted to integrate EBP into their daily work. The participating nurses in observations and focus groups were chosen to give rich information regarding emerging codes and categories, for instance task accomplishment and adjusting knowledge to practice. All focus groups were conducted at the nurses' workplaces and consisted of four to five participants. The focus groups were moderated by ÅR and co‐moderated by SH. They lasted between 55–65 min and were audiotaped and transcribed. The data collection and analysis continued until no new categories emerged, and we determined that theoretical saturation was achieved (Glaser, 1978).

### 2.3 Data analysis

We performed an open analysis of the data from the observations and focus groups in the same analysis, concurrently with the data collection, according to the principles of classical grounded theory using the constant comparative method (Glaser, 1978; Glaser & Strauss, 1967). During the analysis, we could see that one of the clinical nurses' concerns was related to their striving to do the best for the patients based on EBP. We then analysed in depth the data related to the nurses' challenges in EBP integration. The lead researcher wrote memos, which were assumptions about relations between the data, articulated as hypotheses that could be tested in the data (Glaser, 1978). As such this was both an inductive and a deductive approach to the data. In the first step of the analysis, the lead researcher systematically identified the relevant emerged codes from the observations and focus groups using the data from Ward A. Next, the researcher identified the emerged codes from Ward B in the same way. The rest of the research team read transcriptions and field notes as well and the whole group of authors discussed the codes. After finishing the separate coding for the two wards, we analysed the codes and categories for the two wards in relation to each other to explore the challenges in integrating EBP in clinical practice.

### 2.4 Rigour

The use of focus group interviews in grounded theory is less common than the use of individual interviews (Hernandez, 2011). However, data with variety and rich information are recommended in grounded theory (Glaser, 1978, 1998). We consider it a strength that we collected data through both observations and focus groups, endeavouring to perform the data collection and analysis in a manner congruent with grounded theory (Hernandez, 2011). To understand what was happening in the investigated fields, we have endeavoured to stay open in the data analysis and refrain from using preconceived ideas or concepts (Glaser, 2013; Glaser & Strauss, 1967). Throughout the study, we have focused on conceptualizing emerging categories and to be aware of the relationships between the categories. The awareness of these relationships is essential in theoretical sensitivity, which is important in grounded theory (Gibson & Hartmann, 2014; Glaser, 1978).

### 2.5 Ethics

Approval for the study was requested from a Regional Committee for Medical and Health Research Ethics, but the study did not require approval (Reference number 2014/35A). The Data Protection Officer for Research and Quality approved the study (Reference number 2013/17344). The hospital where the study was performed also permitted the study (reference number 201200448‐27). The participants were recruited on a voluntary basis, based on information about the study from their leader and oral and written information from the researcher during the observation period. When the researcher observed the nurse working with the patient, the nurse first informed the patient and obtained oral consent. The researcher recruited the participants to the focus groups in cooperation with the ward leaders, and written consent was obtained.

## 3 FINDINGS

**Figure 2:**
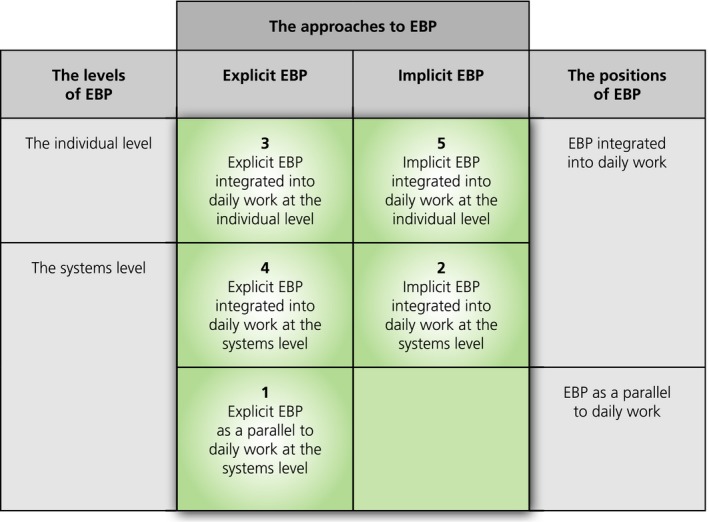
Multidimensional EBP integration framework

This study revealed three significant and interacting dimensions of EBP integration that may help explain the complexities involved when nurses attempt to integrate EBP in their daily practice. The dimensions are as follows: approach to EBP, position of EBP in daily work and organisational level of EBP. By approach, we mean the way of enacting EBP. Two approaches to EBP were identified; explicit EBP (visible and emphasized in the ward) and implicit EBP (invisible and hidden in the background in the daily work in the ward). We also identified two positions of EBP in daily work. With position, we mean how EBP was related to the daily work in the wards. EBP could either be integrated into the daily workflow or it could be performed as a parallel activity to daily work. Finally, we identified two organisational levels of EBP; the systems level and the individual practitioner level. With organisational level, we mean how EBP was integrated into the work at the wards. It could be built into the general routines of the ward, or it could be considered the responsibility of the individual healthcare worker to use EB knowledge when caring for individual patients. The core concept “multidimensional EBP integration” embraces the interactions between these dimensions (Figure 2).

The multidimensional EBP integration framework visualizes five combinations that give meaning based on data in this study. In the next sections, we explore the five observed patterns of EBP integration in further detail.

### 3.1 An explicit EBP as a parallel to daily work at the systems level

The EBP project in Ward A represented the dimensions of an explicit EBP performed as a parallel to daily work at the systems level (i.e. alternative 1, Figure 2). Here, the EBP was visible and articulated. All nurses were involved in discussions regarding EBP and the appropriate knowledge to be used in actual situations, indicating that their attitudes had been influenced and that they were more aware of the knowledge source:

I think that our focus on EBP contributes to a greater awareness of what may be the right thing to do. Not just to find an answer, but to find the right answer for the treatment and for the follow‐up. (Focus group I, SN 4)

This activity running parallel to the nurses' daily work in the ward could be conflicting for the nurses. On the one hand, the nurses appreciated the opportunity to work with EBP and quality improvement on a relevant theme, free from daily duties and together with their colleagues. On the other hand, the nurses encountered difficulty in relating this work to their daily patient work. When the groups finished their project periods, they struggled to put the new evidence to use in the daily work. Even if the project motivated the nurses, they felt that they did not have the power to change practice with a new guideline or just with new evidence. The nurses experienced a strong dependence on the managers and physicians who had to formally approve the new clinical guideline and to accept the new knowledge to be used. The nurses were looking for systems and structures to get new evidence more easily and rapidly incorporated into daily routines.

### 3.2 An implicit EBP integrated into daily work at the systems level

The huddle board programme in Ward B represented the dimensions of an implicit EBP integrated into daily work at the systems level (i.e. alternative 2, Figure 2). The EBP was implied in standardized recommendations and measures integrated directly into daily routines as a part of the nurses' daily tasks. This integration made the nurses comply with the request to use the EB recommendations and measures. However, the research evidence tied to the huddle board target areas was not highlighted in daily work:

I feel that the huddle board in a way has become a visual systematization of things we did already. Everything gets very visible, everyone sees it and it is more organized. We did exactly the same things earlier too, but now it is made visible. (Focus group IV, SN 8)

The individual nurses did what the organization expected them to do to promote patient safety and quality improvement, but they did not consciously relate to the evidence or seem to understand their use of knowledge as EBP. The leaders and teaching nurses in the ward did organize reflection groups for the nurses once a week, discussing professional challenges and clinical problems. As such, they stimulated the nurses' critical thinking and inquiry. Nevertheless, this was not visibly linked to the huddle board target areas.

### 3.3 An explicit EBP integrated into daily work at the individual level

Based on the definition of EBP, the ideal is an explicit EBP integrated into daily work for each individual patient (i.e. alternative 3, Figure 2). In this study, the clinical nurses recognized this ideal and were striving to realize it. Nevertheless, the findings indicated a gap between the ideal and the actual performance of individualized patient care. This gap was related to the challenges of getting new research evidence to be used and the strong emphasis on standardized routines. Due to the latter, the nurses' pattern of behaviour was dominated by filling out checklists, whereas their focus on the needs of each individual patient receded into the background. For instance, the nurses in Ward B referred to the whiteboard as a visual checklist, which they appreciated because of better safeguarding of the risk areas. Simultaneously, they expressed scepticism of the use of checklists because it was challenging to strike the right balance between the risk assessment “check‐offs” and other patient needs for nursing care:

Preventing falls, which is a theme in the huddle board, is part of basic nursing care. Holistic nursing care disappears when filling out the forms. When you have been working for a while, you know what you need to do to prevent falls. I think this [fragmented and task oriented practice] is scary. (Focus group III. AN 6)

### 3.4 An explicit EBP integrated into daily work at the systems level

We could not see an extensive use of an explicit approach to EBP integrated into daily work at the systems level in this study (i.e. alternative 4, Figure 2). Even if some nurses demonstrated their awareness of the knowledge they used, they seldom could refer to where they had gained it:

I am very focused on clinical issues and feel that I update myself reading every new procedure coming in the ward. But there is a lot of information. We mix it with information about the patient and all the things you should remember during the day. You do not think that “this knowledge” I derived from “there”. You use knowledge without knowing exactly where you got it. (Focus group III, RN 2)

### 3.5 An implicit EBP integrated into daily work at the individual level

The combination of the dimensions of an implicit EBP integrated into daily work at the individual level was difficult for the researcher to observe in practice and would be difficult for the nurses to put into words because of its implicitness (i.e. alternative 5, Figure 2). What we could observe was the nurses providing care according to prevailing clinical guidelines at the wards, which indicates integration of EB knowledge. Furthermore, their explicit recognition of the fact that they provided care based on many different sources of knowledge, including new guidelines being introduced, support the idea of an implicit EBP integrated into daily work at the individual level.

## 4 DISCUSSION

This study revealed three interacting dimensions of EBP integration that may explain the complexities and challenges when nurses attempt to integrate EBP in hospital wards. We identified two approaches (explicit EBP and implicit EBP), two positions (EBP integrated into daily work and as a parallel to daily work) and two levels of EBP (the systems level and the individual level). The interactions between the dimensions gave five meaningful combinations in this study. In the following subsections, we have organized the discussion according to the most central findings; challenges regarding EBP as a parallel to daily work, use of standardization and routinization to promote EBP at the systems level and the movement from the systems level to the individual level.

### 4.1 EBP as a parallel to daily work

The findings showed that clinical nurses who applied the explicit approach to EBP as a parallel to daily work increased their awareness of evidence and what might be the right things to do. They wanted to apply new evidence, but at the systems level they did not have the authority to integrate the new knowledge on their own and they lacked an efficient mechanism for ensuring timely integration into their daily work in the ward. This perspective demonstrates challenges well known from the literature; clinical nurses striving to learn EBP and develop EB guidelines but failing to integrate the new evidence (Adib‐Hajbaghery, 2007; Aitken et al., 2011; Pitkänen, Alanen, Rantanen, Kaunonen, & Aalto, 2015; Solomons & Spross, 2011). The lack of organizational structures for adopting new guidelines may be related to an organization's limited capacity for change, which is still a highlighted barrier to EBP integration (Flodgren, Rojas‐Reyes, Cole, & Foxcroft, 2012; Sadeghi‐Bazargani et al., 2014; Solomons & Spross, 2011; Williams, Perillo, & Brown, 2015). We argue that lack of organizational support must be solved by organizational initiatives to create a structure for integration of new EB guidelines. Otherwise, these organizational barriers will impede healthcare professionals' ability to increase and maintain their use of EBP, even if they are motivated and have knowledge about the application of EBP (Williams et al., 2015).

### 4.2 Standardization and routinization may promote EBP at the systems level

Our findings suggest that the implicit approach to EBP integrated into daily work at the systems level could stimulate the nurses' research use, even if the evidence was not highlighted in their daily work. We argue that research use through EB guidelines integrated through a tool such as the huddle board might contribute to improved sustainability of guidelines through persistent routinization of action. This is consistent with other studies suggesting that routinization or normalization increases clinicians' use of guidelines and stimulate guideline sustainability (Fleiszer, Semenic, Ritchie, Richer, & Denis, 2015; May, Sibley, & Hunt, 2014).

However, the implicit approach to EBP represented a challenge because the nurses lacked awareness about the underlying evidence and focused rather on the tool and the standardized observations, registrations and measures. Thus, the nurses used evidence without being conscious of it. This could constitute a possible risk, as excessive routinization may impede a person's ability to detect, interpret and handle contextual changes, thereby sustaining existing patterns of behaviour when change is needed (Ellström, 2006). Furthermore, standardization and routinization could lead to individual patient needs being disregarded. Our findings visualize that a way to succeed in integrating EBP into daily work could be to establish measures at the systems level before one can expect EBP to be established at the individual level. A tool, such as the huddle board sessions combined with measures to make and keep the underlying evidence explicit, may make this possible. We turn to this issue next.

### 4.3 Movement from the systems level to the individual level

A movement from the systems level to the individual level entails moving from a structured approach, where EBP is integrated and EB guidelines are applied in daily work at the ward level, to individualized patient‐tailored care informed by relevant evidence. We argue that this movement could be supported by making EBP explicit and visible at the systems level. This could be achieved by stimulating the clinical nurses' awareness through systematic reflection and discussion about the relevance of risk assessment for the individual patients and by making explicit the research evidence underpinning the EB guidelines. Leaders might gradually integrate research activities into the nurses' everyday routines to change the focus towards valuing research evidence as a way of providing high‐quality treatment and care for individual patients (Scott‐Findlay & Golden‐Biddle, 2005). This implies discussing the relevance of general guidelines for the individual patient. Unless consciously addressed, individualized care could be ousted by EB standardized programmes (Norlyk, Haahr, Dreyer, & Martinsen, 2017). Patient centeredness and individualized care are necessary to achieve EBP in specific clinical situations (Brown, 2014; Melnyk & Fineout‐Overholt, 2015). A tool such as the huddle board sessions could be a stepping stone to focusing on individual patient situations through combining the standardized risk assessments for individual patients with the integration of patient preferences in clinical problem solving. Leadership may contribute to increased patient‐centred care by being close to care delivery, by teaching and supervising clinicians and by addressing how quality improvement and EBP relate to the care of individual patients (Lalleman, Smid, Dikken, Lagerwey, & Schuurmans, 2017). Giving the clinical nurses and their ward leaders the opportunity to discuss and integrate research evidence into the nurses' everyday routines and into the care of individual patients may stimulate the nurses to value and probably use the research findings (Scott‐Findlay & Golden‐Biddle, 2005).

### 4.4 Strengths and limitations

By using grounded theory methodology, we have been able to develop a theoretical perspective and framework that captures the dimensions of integrating EBP into daily work. This framework highlights the challenges involved in attempting to integrate EBP into the daily work of nurses by illuminating how the dimensions interact. Data gave few indications that a sixth combination; an explicit EBP as a parallel to daily work at the individual level occurred in this study, although this would easily be envisioned as a possibility. Due to time constraints, we did not have the possibility to investigate this issue further, although we recognize that it could have strengthened the richness of the findings.

## 5 CONCLUSIONS AND IMPLICATIONS

This study revealed a multidimensional EBP integration framework. The framework visualizes the complexity in clinical nurses' daily work and the efforts that need to be put in to achieve EBP integration.

This new perspective on the dimensions of EBP integration may have implications for clinical practice and probably could also be a guide for further research. The first objective could be to establish a structure to support EBP with an appropriate tool at the systems level. In such structures, EB guidelines developed by nurses as a parallel to daily work may be easier to apply. Furthermore, organizational and individual initiatives are important steps towards making the evidence in the EB guidelines visible to the nurses in clinical patient situations.

For further research and development of the multidimensional EBP integration framework, we recommend studying more hospital wards in the clinical nurses' daily work. As shown in this study, research use through EB guidelines in the implicit approach to EBP integrated into daily work might contribute to improved sustainability of guidelines. This could be appropriate for further research using a tool such as a huddle board and conducting a study of participants primarily using an explicit approach to EBP integrated into daily work at the systems level to integrate EB guidelines in clinical practice.

## CONFLICT OF INTEREST

The authors have declared no conflict of interest.

## AUTHOR CONTRIBUTIONS

All authors have agreed on the final version and meet at least one of the following criteria [recommended by the ICMJE (https://www.icmje.org/recommendations/)]: (a) substantial contributions to conception and design, acquisition of data or analysis and interpretation of data; (b) drafting the article or revising it critically for important intellectual content.
